# Mapping Educational Outcomes and Implementation Challenges of Problem-Based Learning Versus Traditional Didactic Teaching in Physiotherapy Curricula: A Scoping Review

**DOI:** 10.7759/cureus.112973

**Published:** 2026-07-19

**Authors:** Rupali Shevalkar, Bharati Asgaokar, Khyati Kothary, Dhvani Shah

**Affiliations:** 1 Physiotherapy School and Centre, Topiwala National Medical College and Bai Yamunabai Laxman Nair Charitable Hospital, Mumbai, IND; 2 Department of Musculoskeletal Physiotherapy, K.J. Somaiya College of Physiotherapy, Mumbai, IND; 3 Department of Cardiorespiratory Physiotherapy, K.J. Somaiya College of Physiotherapy, Mumbai, IND

**Keywords:** clinical decision-making, curricular integration challenges, pedagogical effectiveness, physical therapy curricula, traditional didactic teaching (tdt), problem-based learning (pbl)

## Abstract

Physiotherapy education is undergoing significant changes to address the growing complexity of clinical practice, leading to a shift from traditional didactic teaching (TDT) to learner-centred methods, such as problem-based learning (PBL). Traditional approaches focus on structured lectures and memorization, whereas active learning emphasizes the clinical context and learner autonomy. This scoping review examines and synthesises the global peer-reviewed literature comparing PBL and TDT in physical therapy curricula to thoroughly assess cognitive outcomes, student engagement, and implementation dynamics. Using the Arksey and O’Malley framework and PRISMA-ScR guidelines, a systematic search of multiple databases was conducted for English-language peer-reviewed articles published between January 1, 2000, and December 31, 2025. Studies included were those that analysed undergraduate or postgraduate physiotherapy groups with direct comparisons between problem-based interventions and traditional lecture controls. Data extraction and thematic synthesis focused on academic performance, clinical reasoning, soft-skill development, student motivation, faculty perspectives, and implementation realities. Eleven studies met the criteria, covering diverse geographic areas and including quantitative, qualitative, mixed-methods research, and review designs. The findings reveal distinct structural differences between the two teaching paradigms. Traditional methods effectively deliver foundational medical knowledge and prepare students for recall-based assessments, but suffer from rapid knowledge loss after examinations. In contrast, PBL enhances long-term retention, critical thinking, clinical reasoning, and communication skills in clinical settings. Active learning also boosts intrinsic motivation and self-directed learning, aligning with students' practical learning preferences. However, the shift to active methods can cause student distress, such as confusion and anxiety, owing to open-ended clinical scenarios. Faculty acknowledge the value of PBL but express concerns about administrative time, loss of syllabus control, and the need for ongoing training of facilitators. Implementation challenges include resistance to curricular change, resource demands, and rigid assessment rubrics. Successful integration is supported by strong leadership, dedicated technology, and faculty development. To address these challenges, modern programs have adopted hybrid models that combine active, team-based modules with traditional elements. Ultimately, PBL and TDT offer complementary strengths in physiotherapy education. A blended curriculum optimizes knowledge acquisition and clinical readiness; however, sustainable implementation requires institutional support, student orientation, and curriculum alignment. Future research should explore the long-term impact of these models on clinical skills.

## Introduction and background

The landscape of physiotherapy education is undergoing a profound transformation, reflecting broader changes in health professions education. With the increasing complexity of clinical practice, traditional pedagogical methods that rely heavily on didactic lectures have been critically examined [1]. There is growing recognition that effective physiotherapy education must not only transmit foundational knowledge but also cultivate essential professional competencies, such as critical thinking, clinical reasoning, effective communication, teamwork, and lifelong learning [2]. In this context, there has been growing interest in educational strategies that prioritise the learner, with problem-based learning (PBL) being a key focus [3].

Traditional didactic teaching (TDT), which has long dominated physiotherapy and other health-related curricula, is characterised by instructor-led lectures, standardised content delivery, and assessment through summative examinations [4]. This approach ensures consistency, content coverage, and scalability but has been criticised for fostering surface learning, passive engagement, and limited clinical readiness. Therefore, physiotherapy education requires methods that promote deeper learning, integration of knowledge, patient-centred decision-making, and interdisciplinary collaboration [5].

PBL, introduced in medical education in the 1960s at McMaster University, offers a compelling alternative to traditional learning methods. It is an instructional approach grounded in constructivist learning theory, wherein students learn through engagement with complex real-world problems in a collaborative environment [3]. In PBL, learning is self-directed, contextual, and student-driven. Facilitators act as guides rather than content experts, encouraging students to identify their own learning needs, gather and analyse information, and apply it to solve clinical problems. This model fosters the development of cognitive and interpersonal skills that are critical in clinical practice, including hypothesis generation, evidence-based reasoning, and reflective thinking [6].

Within physiotherapy education, the implementation of PBL has been uneven across institutions and regions. Some programs have fully embraced PBL curricula, while others have partially integrated it or maintained a primarily didactic approach. Despite increasing adoption, the evidence comparing the effectiveness of PBL and TDT in physiotherapy education remains heterogeneous [7]. Some studies have suggested that PBL enhances student motivation, engagement, and clinical decision-making abilities. Others have argued that traditional lectures remain efficient and effective, particularly in conveying foundational knowledge. This divergence in findings highlights the need for a comprehensive synthesis of the existing literature to guide curriculum development [5,8].

Moreover, although PBL is theoretically aligned with the goals of physiotherapy education, its implementation presents practical challenges. These include the need for faculty training in facilitation, increased resource demands, adaptation of assessment methods, and resistance to change from educators and students accustomed to conventional teaching [2]. These factors can influence the success and sustainability of PBL initiatives and must be considered in any evaluation of their effectiveness.

Physiotherapy, as a discipline, occupies a unique position in health professions education. It demands both cognitive and psychomotor skills, requires the integration of biomedical knowledge with humanistic and behavioural competencies, and relies heavily on the therapist-patient relationship [9,10]. It is essential to assess the impact of teaching methods on knowledge, professional identity, and clinical competence. Despite extensive PBL research in medicine and nursing, physiotherapy-specific evidence is limited, underscoring the need to align methods with educational goals.

In recent years, health education regulatory bodies and accreditation agencies have emphasized outcome-based education and competency-based curricula. These shifts have placed additional pressure on physiotherapy educators to adopt and evaluate teaching methods that transmit information and produce competent, reflective graduates who can adapt to changing environments [11]. Consequently, it is crucial to examine how PBL and TDT impact educational outcomes relevant to physiotherapy, including academic performance, clinical reasoning, communication, collaboration, and readiness for practice.

Several systematic and scoping reviews have investigated the impact of PBL on medical education, often reporting favourable outcomes in terms of student satisfaction and higher-order cognitive skills [12]. The findings regarding academic performance and clinical competence are inconsistent. In physiotherapy, PBL research is growing but is fragmented and context-dependent, highlighting the need to consolidate evidence to clarify the strengths and limitations of each approach.

This scoping review aims to fill this gap by systematically mapping the literature comparing PBL and TDT in physiotherapy education. This study seeks to answer the following questions: (1) What are the comparative educational outcomes of PBL and TDT in physiotherapy curricula? (2) What challenges and facilitators are associated with implementing PBL in physiotherapy education?

By addressing these questions, this review offers a comprehensive understanding of how different pedagogical approaches influence the development of competent physiotherapy professionals. It also highlights key areas for future research, including long-term clinical outcomes, assessment strategies, and faculty development needs. Furthermore, the findings may guide curriculum development by encouraging the integration of learner-centred approaches with traditional teaching methods while emphasizing the importance of educational policies that support faculty training and effective implementation of these strategies.

When choosing a scoping review methodology, we acknowledge the complexity and variability of the evidence. Unlike systematic reviews that assess intervention effectiveness using strict inclusion criteria, scoping reviews are well-suited for examining broad topics with diverse study designs and outcome measures. Following the framework of Arksey and O’ Malley [13] and further refined by Levac et al. [14], this review systematically identifies, charts, and synthesises existing evidence, highlighting gaps in the literature and proposing future directions.

This review aims to compare the strengths, limitations, and contextual applicability of PBL and TDT in physiotherapy education, supporting evidence-based curriculum reform aligned with evolving global and interdisciplinary healthcare demands. 

Objective and review questions

This review was guided by two primary research questions: (1) What are the comparative educational outcomes of PBL and TDT in physiotherapy education? (2) What are the challenges and facilitators associated with the implementation of PBL in physiotherapy curricula? These questions were framed to guide the identification, selection, and synthesis of relevant literature addressing the effectiveness and practical implications of pedagogical strategies in physiotherapy education.

## Review

Method

This scoping review aimed to systematically explore and synthesise the literature comparing PBL and TDT in physiotherapy education. A scoping review methodology was selected because of its appropriateness for mapping broad research fields, identifying key concepts, and highlighting knowledge gaps in diverse and heterogeneous bodies of literature. The methodology was informed by the framework proposed by Arksey and O'Malley [13], refined through enhancements suggested by Levac et al. [14], and aligned with the Preferred Reporting Items for Systematic Reviews and Meta-Analyses Extension for Scoping Reviews (PRISMA-ScR) guidelines [14].

A comprehensive search strategy was developed in collaboration with a health science librarian. The search was conducted across five major electronic databases to ensure broad coverage of the relevant literature: PubMed, Cumulative Index to Nursing and Allied Health Literature (CINAHL), Web of Science, and Scopus.

The databases were searched using a combination of controlled vocabulary (e.g., MeSH terms) and keywords related to PBL, TDT, and physiotherapy education.

An example of a search strategy for PubMed is as follows: "Problem-Based Learning"[Mesh] OR "problem-based learning"[tiab] OR "PBL"[tiab] OR "active learning"[tiab] OR "case-based learning"[tiab]) AND ("Physical Therapy Specialties"[Mesh] OR "Physical Therapy Department, Hospital"[Mesh] OR "physiotherapy"[tiab] OR "physical therapy"[tiab] OR "physiotherapists"[tiab] OR "physical therapists"[tiab]) AND ("Curriculum"[Mesh] OR "Education, Professional"[Mesh] OR "Educational Measurement"[Mesh] OR "education"[tiab] OR "curriculum"[tiab] OR "pedagogy"[tiab] OR "academic performance"[tiab] OR "clinical reasoning"[tiab] OR "implementation barriers"[tiab]).

The search was limited to studies published in English from January 2000 to December 2025 to reflect contemporary educational practices. Additional records were identified by manually searching the reference lists of key articles and relevant review papers. To ensure relevance and consistency, a set of inclusion and exclusion criteria was developed using the population-concept-context (PCC) framework recommended for scoping reviews.

Inclusion Criteria

Studies were selected for inclusion in this scoping review if they met the following criteria: First, the studies focused directly on physiotherapy education, encompassing both undergraduate and postgraduate student cohorts. Second, the research must have explicitly compared PBL and TDT methods across any aspect of physiotherapy education, such as academic performance, critical thinking, clinical reasoning, and communication skills. Third, only articles published in peer-reviewed journals were considered. Fourth, this review considered multiple research methodologies, including qualitative, quantitative, mixed-methods designs, and reviews. Finally, the search was temporally bounded to studies published between January 1, 2000, and December 31, 2025.

Exclusion Criteria

Studies were excluded from the review if they met any of the following criteria. First, research involving other allied health professions, such as nursing, medicine, or pharmacy, was excluded if it lacked specific or separate subgroups and analyses for physiotherapy. Second, non-comparative studies were excluded if they focused solely on an isolated PBL or TDT curriculum without a direct contrast between the two models. Third, grey literature, including conference abstracts, editorials, letters to the editor, academic theses, and unpublished dissertations, was excluded to maintain high peer-reviewed quality. Fourth, studies were excluded if their full texts were completely unavailable through institutional subscriptions or open-access repositories.

Study Selection

All search results were imported into EndNote reference management software to remove duplicates. The titles and abstracts of the remaining studies were independently screened by two reviewers. Full-text screening of potentially relevant articles was conducted to assess eligibility based on the inclusion and exclusion criteria.

Disagreements between the reviewers during the screening stage were resolved through discussion or consultation with a third reviewer. A PRISMA flow diagram was used to document the study selection process, including the number of records identified, screened, excluded, and included, with reasons for exclusion provided at the full-text screening stage [15].

Data Extraction and Charting

To ensure systematic and comprehensive mapping of the literature, a standardized data extraction form was developed in Microsoft Excel (Microsoft® Corp., Redmond, WA) and pilot-tested on a sample of five studies to confirm its clarity and applicability. The following specific items were extracted from each included study: author(s), publication year, country of origin, and study design (qualitative, quantitative, mixed-methods, or review). Additionally, the extraction form captured the educational level (undergraduate or postgraduate), overall sample size, and participant demographics, along with a detailed description of both the PBL and TDT interventions evaluated. Finally, the reviewers extracted the specific outcomes measured (such as academic performance, clinical reasoning, and student satisfaction), the main findings and conclusions, the reported barriers and facilitators to implementation, and any applicable quality appraisal. To safeguard the validity and accuracy of the extracted data, the extraction process was independently conducted by two reviewers, and any discrepancies were resolved through mutual consensus.

Quality Assessment

Although quality appraisal is not mandatory in scoping reviews, we conducted a descriptive assessment of study quality using appropriate tools for different study types to provide context for the interpretation of the findings. The Joanna Briggs Institute (JBI) Critical Appraisal Tools were used for quantitative studies, and the Critical Appraisal Skills Program (CASP) checklist was applied for qualitative studies [16].

The results of the quality assessments were not used to exclude studies but were reported to inform the discussion of evidence strength and methodological limitations.

Data Synthesis and Analysis

A thematic synthesis approach was employed to analyse and interpret the extracted data. Following initial charting of the included studies, recurring themes and categories were identified through inductive coding and grouped into six primary domains: academic and cognitive outcomes, clinical reasoning and decision-making, communication and interpersonal skills, student engagement and motivation, faculty perceptions and institutional factors, and barriers and facilitators to implementation. Within this framework, both convergent findings supported across multiple studies, and divergent insights stemming from contextual or structural variations were systematically documented. Ultimately, these synthesized findings are presented narratively and supplemented with structured summary tables to illustrate the comparative differences between the PBL and TDT approaches.

Ethical Considerations

This review analysed publicly available data from published studies and did not involve human participants; therefore, institutional ethical approval was not required.

Limitations of the Methodology

Although every effort was made to conduct a comprehensive and rigorous review, certain limitations must be acknowledged. The search was limited to English-language publications, which may have introduced a language bias. Additionally, grey literature, including theses and reports, was not included. Heterogeneity in study designs and outcome measures also limited the ability to draw direct comparisons or perform a meta-analysis.

**Figure 1:**
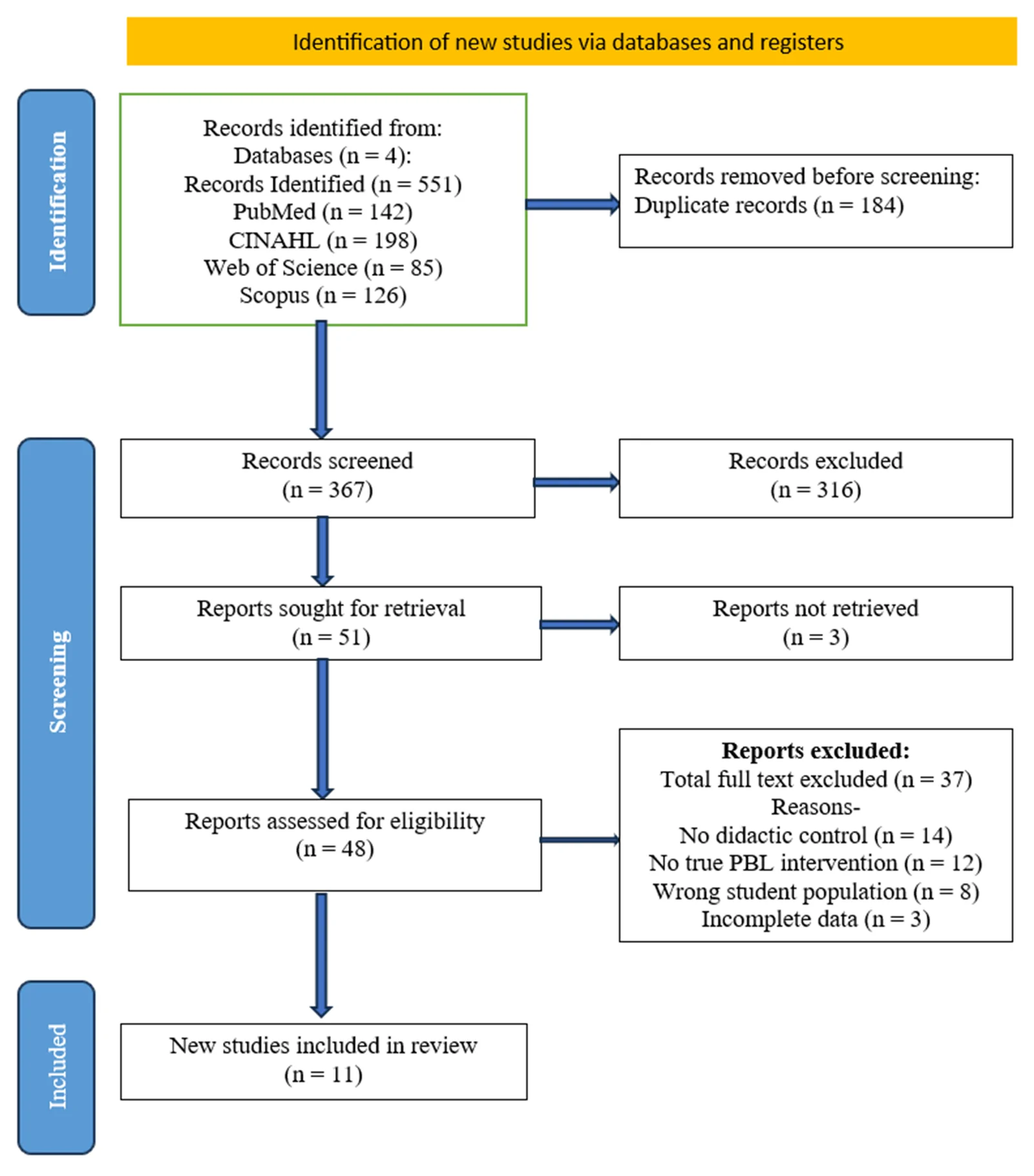
PRISMA flow diagram of the search and selection processes

Figure 1 presents the PRISMA flowchart of the study selection process [15].

Results

Study Selection and Characteristics

Following the PRISMA-ScR framework, the initial multi-database search across PubMed, CINAHL, Web of Science, and Scopus yielded 551 unique records published between 2000 and 2025. After removing 184 duplicate citations, 367 records were screened based on their titles and abstracts. This phase excluded 319 records that did not align with the target population or pedagogy of the study.

Of the 51 reports sought for retrieval, three full-text articles were unavailable because of institutional access barriers. A total of 48 full-text reports were assessed for eligibility based on the predefined inclusion criteria. Thirty-seven articles were excluded for the following reasons: absence of a didactic comparator (n = 14), lack of a true PBL intervention (n = 12), inappropriate study population (n = 8), and incomplete data (n = 3). Ultimately, 11 peer-reviewed studies were included in the final analysis (Figure 1).

The included studies demonstrated substantial methodological diversity, comprising quasi-experimental comparative designs (n = 3) [17-19], retrospective or non-randomised cohort studies (n = 2) [20,21], mixed-methods investigations (n = 3) [22-24], and one conceptual framework study (n = 1) [10]. In addition, two systematic or scoping reviews were included (n = 2) [25,26], collectively reflecting a broad range of evidence sources used to map the educational outcomes and implementation aspects of PBL and TDT.

Themes Identified

Academic performance: Evidence indicates that TDT and active learning approaches yield comparable outcomes in short-term factual knowledge acquisition, as reported in systematic and narrative reviews [10,26]. However, PBL demonstrates superior long-term knowledge retention [26].

Primary studies further suggest that active and PBL models significantly enhance academic performance and theoretical examination scores compared to traditional teaching [17,23]. In contrast, other comparative studies have reported no significant differences in overall academic outcomes between the PBL and TDT approaches, indicating that active learning methods can serve as effective alternatives without compromising foundational knowledge [19,20].

Clinical reasoning and critical thinking: Multiple comparative studies have highlighted that PBL and student-centred approaches significantly enhance clinical reasoning and critical thinking skills [17,18,23]. These models promote deeper cognitive engagement, knowledge integration, and analytical thinking compared to traditional didactic instruction.

Conversely, TDT methods appear less effective in fostering higher-order cognitive skills, aligning with active approaches primarily in terms of short-term recall outcomes [26].

Communication and soft skills: Active learning approaches contribute substantially to the development of communication, teamwork, and professional competencies [10]. A narrative review by Solomon [10] and a systematic review [26] report that PBL enhances self-directed learning readiness, communication skills, and adaptability in clinical environments.

A retrospective cohort study demonstrated that students trained in PBL or hybrid curricula performed significantly better in clinical skills during internships than those in traditional programs [21]. However, some studies have indicated equivalent improvements in self-efficacy and clinical performance across both active and traditional teaching methods [20].

Student engagement and motivation: Active learning techniques enhance student engagement by leveraging psychological principles, although transitioning to these methods can be challenging for educators. A future-oriented study utilising the R-SPQ-2F tool revealed that the inclusion of PBL modules boosts intrinsic motivation, encouraging students to move away from rote memorisation and towards understanding cases in depth [24]. Similarly, evidence suggests that PBL enhances engagement, confidence, and evidence-based practice competency [23]. However, challenges during implementation have been reported, including student anxiety, role confusion, and a preference for structured lectures [23]. While systematic evidence indicates overall satisfaction with PBL [26], variability in students’ experiences persists.

Faculty perspective: The transition to student-centred learning increases the faculty’s workload and requires substantial preparation. A scoping review highlighted the significant institutional and resource-related demands associated with PBL implementation [10].

Additionally, effective outcomes depend heavily on facilitator expertise and adherence to structured delivery, as inconsistencies in the tutor’s performance can influence the results [26].

Implementation barriers and facilitators: The implementation of PBL and related approaches is constrained by institutional and logistical challenges. Reviews have identified key barriers, including limited resources, inadequate infrastructure for small-group learning, and rigid evaluation systems [10,25].

Primary studies further emphasise challenges such as insufficient faculty training, large class sizes, and student resistance to active learning models [17,22].

Facilitators include structured orientation programs to prepare students for PBL environments [22] and the adoption of blended or hybrid teaching models to balance curriculum coverage with active engagement [21,23].

To conduct a systematic analysis of the literature, the chosen articles were organized into a detailed summary matrix (Table 1). The studies were sorted by methodology, publication year, and research design, such as quasi-experimental or qualitative approaches, to enable comparisons across studies and identify common patterns and trends. Subsequently, each publication was meticulously reviewed to determine its main objectives, key findings, and any limitations in implementation for inclusion in the final table of the review.

**Table 1:** Summary of the included studies

Authors, Year	Study Design	Purpose	Key Mapped Findings	Methodological/Implementation Limitations
Van Duijn and Bevins, 2005 [21]	Retrospective comparative cohort study	To compare the clinical internship performance of physical therapy students trained in problem-based learning (PBL), traditional, and mixed-model curricula.	Students from PBL and mixed-model curricula scored significantly higher in clinical performance (such as examination and evaluation skills) during internships than those from traditional lecture-based tracks.	Dependent on clinical instructors' subjective evaluations via the CPI tool. Retrospective design makes it difficult to control for individual baseline student characteristics or varying clinic environments.
Solomon, 2005 [10]	Critical narrative and scoping review	To synthesize global evidence on PBL outcomes and identify operational issues relevant to integrating it into physical therapy programs.	While factual knowledge acquisition is equivalent to traditional lectures, PBL yields superior outcomes in communication, self-directed research readiness, and clinical placement adaptability.	Lacks a structured systematic scoring framework for data pooling. Highlights major institutional implementation bottlenecks, specifically intensive resource demands, small-group space limits, and heavy faculty workload.
Kasai et al., 2006 [19]	Comparative quasi-experimental study	To compare the short-term educational effectiveness of PBL versus traditional lecture-based teaching in undergraduate physiotherapy education.	No significant difference in overall examination scores between PBL and lecture groups. The PBL group showed enhanced SDL and consistent performance.	Small sample, non-randomized, short-term, single-centre.
O'Donoghue et al., 2011 [26]	Systematic review of controlled trials	To evaluate the effectiveness of PBL versus traditional lecture methods in entry-level professional therapy education.	Found no significant differences between PBL and traditional lectures regarding short-term factual knowledge. However, PBL consistently demonstrated superior long-term retention, clinical performance, and student satisfaction.	Confounded by poor implementation fidelity and variable tutor behaviours across studies. Most included trials relied heavily on subjective self-reporting rather than objective, blinded clinical measures.
Keiller and Louw, 2013 [24]	Mixed-methods descriptive study	To evaluate undergraduate physiotherapy students' study approaches within a newly integrated PBL curriculum module.	Utilized the R-SPQ-2F tool. Found a statistically significant baseline preference for deep learning over surface styles. As the module progressed, students showed an increase in intrinsic motivation and actively shifted from surface rote habits to deep, case-applicable understanding.	Single-cohort study at one institution restricts broad geographical generalizability. Relies heavily on immediate, mid-year self-reported data metrics without tracking the long-term sustainability of these learning shifts.
Castro-Sánchez et al., 2012 [18]	Quasi-experimental comparative study (non-randomized, multi-centre academic cohort)	To compare learning strategies and study preferences between physiotherapy students exposed to PBL versus conventional lecture-based teaching.	PBL significantly enhanced deep learning strategies, including organization, integration of ideas, and collaborative learning, while reducing surface learning behaviours such as rote memorization, lack of purpose, and fear of failure; no strong evidence of superior objective academic performance despite improved learning approaches.	Non-randomized design with potential selection bias; reliance on self-reported learning inventories (CLSI, ASSIST) rather than objective performance outcomes; lack of clinical follow-up or long-term competency assessment; heterogeneity across institutions and teaching contexts may affect internal validity.
Lennon et al., 2019 [23]	Mixed-methods, quasi-experimental	This study evaluated whether implementing PBL in an undergraduate physiotherapy programme improves students' early evidence-based practice (EBP) competencies compared with prior traditional lecture-based teaching.	Significant improvements in EBP knowledge, confidence, and student engagement with PBL compared to traditional didactic teaching (TDT).	Non-randomized design, historical control, self-reported outcomes, single-centre study, lack of long-term follow-up.
van Lankveld et al., 2019 [20]	Non-randomized experimental pre-post study	To compare educational outcomes and self-reported self-efficacy between self-directed learning (SDL) and instruction-based learning (IBL) in physical therapy education.	Both groups yielded equivalent results in theoretical knowledge and clinical physical therapy performance. Professional self-efficacy increased equally in both cohorts over time, indicating that active self-direction serves as a viable curriculum alternative.	Non-randomized assignment limits internal validity. Sizable baseline attrition occurred in survey participation (62% enrolment response), and long-term habits essential to lifelong learning were not tracked longitudinally.
Pathmanathan et al., 2022 [25]	Systematic review	Evaluate effectiveness of PBL versus traditional teaching in physiotherapy education.	PBL improves EBP, learning strategies, and student preference; evidence limited.	Very small evidence base, high bias, heterogeneity, no meta-analysis, preprint status.
Kerry et al., 2024 [22]	Mixed-methods exploratory study	To evaluate student expectations, intrinsic readiness, and psychological transitions during a large-scale institutional shift to a PBL curriculum.	Initial student expectations were highly positive. However, actual implementation triggered role confusion, anxiety over case ambiguity, and a strong preference for lecture-based predictability.	Single-centre design limits generalizability. Identifies deep-seated student resistance to abandoning rote learning and highlights an urgent need for pre-PBL orientation.
Elgheit and Nashat, 2025 [17]	Mixed-methods prospective cohort study	To evaluate the integration of case-based active learning within undergraduate applied physiology modules.	Active, case-based learning significantly improved academic performance and clinical reasoning compared to traditional lecturing. It successfully bridged the gap between basic sciences and clinical practice while increasing student.	Single-cohort focus lacks long-term evaluation of knowledge retention. Implementation required substantial faculty preparation time and ran into logistical constraints with large class sizes.

Discussion

This scoping review thoroughly examined the literature on PBL and TDT in physiotherapy education. By analysing 11 studies, this review uncovers important findings regarding the influence of these teaching methods on various aspects of student learning and development, such as academic performance, clinical reasoning, communication skills, student engagement, faculty opinions, and practical application. A significant observation from this review is related to academic performance, which is a crucial element in educational evaluation. Although limited research has focused on this aspect, the findings have been mixed. Multiple studies have consistently reported no significant differences in immediate examination performance between PBL and traditional lecture-based approaches [19,20,26]. Evidence suggests that PBL effectively retains fundamental theoretical knowledge, even though it involves less direct teaching than traditional methods. However, a key strength of PBL lies in its ability to enhance deeper learning processes and develop professional competencies. Comparative and quasi-experimental studies have demonstrated that PBL significantly promotes self-directed learning, intrinsic motivation, and the adoption of deep-learning strategies over surface approaches [18,24]. Similarly, Lennon et al. (2019) reported improved EBP knowledge, confidence, and student engagement in PBL cohorts compared with traditional teaching [23]. These findings are further supported by systematic reviews indicating superior long-term retention, clinical reasoning, and student satisfaction associated with PBL [25,26].

Perhaps the most compelling evidence in favour of PBL lies in its effects on clinical reasoning and critical thinking [6,27]. This domain was assessed using tools such as the Health Sciences Reasoning Test (HSRT) and Script Concordance Test (SCT), indicating that PBL significantly enhances students’ diagnostic and decision-making abilities. These competencies are essential in physiotherapy, where practitioners must synthesize patient history, clinical findings, and therapeutic knowledge to develop effective treatment plans. PBL emphasises inquiry, self-directed learning, and problem-solving, which directly support the development of these skills. In contrast, TDT has often been criticised for fostering rote memorisation without promoting higher-order thinking or adaptability in dynamic clinical environments [28].

PBL enhances clinical readiness and performance. Van Duijn and Bevins found that students who participated in PBL and hybrid curricula outperformed those in traditional programs during their clinical internships [21]. This supports the broader evidence that PBL helps students apply theoretical knowledge in clinical settings, improving their decision-making and patient care skills.

Communication and interpersonal abilities were also identified as significant and distinguishing factors. The group-oriented nature of PBL naturally encourages active listening, the provision of constructive feedback, and a sense of shared responsibility, all of which are crucial in multidisciplinary clinical teams [29]. Additionally, students' qualitative feedback emphasized a feeling of personal development and greater confidence in articulating their thoughts and ideas. Conversely, TDT is often characterized as being instructor-focused, offering limited opportunities for student interaction or peer involvement, which could impede the cultivation of soft skills essential for clinical practice.

Similar patterns emerged in the areas of student engagement and motivation as well. Learners in PBL environments exhibit higher intrinsic motivation, enjoy the learning process more, and experience a stronger sense of ownership over their educational paths. PBL was characterized as “authentic,” “relevant,” and “stimulating,” indicating its closer resemblance to the realities of clinical practice than other approaches. Students valued active engagement in PBL, which involved researching, discussing, and applying concepts in context, rather than passively absorbing information [30]. Nonetheless, some students initially struggled with the self-directed aspect of PBL, particularly in the absence of adequate guidance. This highlights the necessity of designing effective support systems to assist students in adapting to more autonomous learning roles in the future. Conversely, students in TDT settings frequently report feelings of boredom, challenges in maintaining attention during lengthy lectures, and a perceived disconnect from clinical practice [31].

The perspectives of the faculty members discussed in this review offer valuable insights into the adoption and maintenance of PBL. While many educators endorse this method for promoting active learning and student autonomy, there are concerns about the significant time and effort required to organise and conduct PBL sessions [32]. Numerous studies have pointed out the growing need for resources, such as increased faculty workload, the requirement for skilled facilitators, and limitations in infrastructure, including spaces for small group learning [10,17]. Students' experiences with PBL can differ, with some encountering initial anxiety, unclear roles, and a preference for traditional lectures at the start of the transition [22]. These challenges highlight the necessity for comprehensive faculty training, student orientation, and gradual curriculum integration. New instructors have reported difficulties in adapting to their roles as facilitators, indicating a significant learning curve for them. Additionally, institutional resistance and rigid curricular structures have been recognised as obstacles to the full implementation of PBL [33]. Nonetheless, factors such as supportive leadership, policy changes, curriculum mapping, and ongoing feedback mechanisms have been consistently linked to successful PBL integration in physiotherapy education [22]. Many institutions have implemented hybrid models that combine PBL and TDT to balance the requirement for foundational knowledge with the advantages of applied and student-centred learning [34]. These models offer flexibility and are particularly effective in facilitating the transition from preclinical to clinical education.

Over the past decade, the rise in the number of publications has signified both a call for reform and difficulties in assessing educational outcomes across different cultural and institutional landscapes. This trend also emphasises the global understanding that physiotherapy, much like other health professions, necessitates not only technical skills but also the ability to think critically, communicate clearly, and make sound professional judgments [35].

Ultimately, the success of any teaching method depends not only on its theoretical foundation but also on the context in which it is implemented, the support available to both students and faculty, and the alignment between teaching strategies and learning outcomes. As physiotherapy continues to evolve in response to complex healthcare challenges, educational institutions must prioritize adaptability, innovation, and improvement [36]. Future research should explore long-term outcomes, such as professional behaviour, patient satisfaction, and career progression, to better understand how different educational approaches shape the future generations of physiotherapists.

In summary, these findings emphasize the need for pedagogical pluralism rather than binary choices in teaching methods research. TDT remains effective for delivering structured content and preparing students for examinations, particularly in the early phases of training. PBL offers distinct advantages in promoting critical thinking, clinical application, and soft skill development. A blended approach tailored to students’ needs and aligned with institutional capacity may represent the most effective model for physiotherapy education [37].

## Conclusions

This scoping review highlights that while TDT remains effective for delivering foundational knowledge, the included studies suggest that PBL may support the development of clinical reasoning, communication skills, and student engagement in physiotherapy education and training. The available evidence indicates that blended approaches may leverage the complementary strengths of both methods. The successful implementation of these approaches appears to depend on faculty readiness, institutional support, and curriculum alignment. Further research is warranted to explore their impacts on clinical competence, long-term learning outcomes, and patient care outcomes.
